# Housing as a Social Determinant of Health in Singapore and Its Association with Readmission Risk and Increased Utilization of Hospital Services

**DOI:** 10.3389/fpubh.2016.00109

**Published:** 2016-05-30

**Authors:** Lian Leng Low, Win Wah, Matthew Joo Ng, Shu Yun Tan, Nan Liu, Kheng Hock Lee

**Affiliations:** ^1^Singapore General Hospital, Singapore; ^2^Family Medicine, Duke-NUS Medical School, Singapore; ^3^Saw Swee Hock School of Public Health, National University of Singapore, Singapore; ^4^Centre for Quantitative Medicine, Duke-NUS Medical School, Singapore

**Keywords:** social determinant of health, readmission, hospital utilization, housing

## Abstract

**Background:**

Residence in public rental housing is an area-level measure of socioeconomic status, but its impact as a social determinant of health in Singapore has not been studied. We therefore aimed to examine the association of public rental housing with readmission risk and increased utilization of hospital services in Singapore.

**Methods:**

We conducted a retrospective cohort study using retrospective 2014 data from Singapore General Hospital’s electronic health records. Variables known to affect readmission risk and health-care utilization were identified *a priori* and include patient demographics, comorbidities, health-care utilization in the preceding 1 year and clinical variables from the index admission in 2014. Multivariate logistic regression was used to evaluate public rental housing as an independent risk factor for admission risk, emergency department (ED), and specialist outpatient clinic attendances.

**Results:**

A total of 14,457 unique patients were analyzed, and 2,163 patients (15.0%) were rental housing residents. Rental housing patients were significantly more likely to be male; required financial assistance; have chronic obstructive pulmonary disease; usage of anti-depressant and anti-psychotic medications; longer length of hospital stay during the index admission; and higher Charlson Comorbidity Index scores. After adjusting for demographics and clinical variables, staying in public rental housing remained an independent risk factor for readmission within 15 and 30 days, frequent hospital admissions and ED attendances in Singapore.

**Conclusion:**

Our study showed an association between public rental housing with readmission risk and increased utilization of hospital services in Singapore. A deeper understanding of the residents’ social circumstances and health seeking behavior would be insightful.

## Introduction

Low socioeconomic status (SES) is well recognized as an independent risk factor for various adverse health outcomes, such as readmission risk (1–3) and hospital utilization (4). Differences in SES contribute significantly to variations in readmission rates between hospitals (5, 6). Most studies evaluating the role of SES have concentrated on individual-level measures of SES, such as educational level, income inequality (7), employment status, or requirement for financial assistance (2). Unfortunately, such detailed information on SES are rarely captured as part of routine clinical care. This limits their utility in estimating readmission risk or planning interventions for high risk patients for whom such information is often unavailable.

Health is determined by a complex interaction between individual characteristics, lifestyle, and the physical, social, and economic environment (8). The interaction of a person’s health with the health system and the community is also well documented in Wagner’s chronic care model (9). The aging population and high prevalence of chronic diseases in many countries had resulted in rapidly escalating demand for hospital services. There is increasing acceptance that adopting a population health approach that promotes healthy communities beyond the hospital setting is an essential component of the new paradigm (10). Some preliminary work on area-level or neighborhood SES measures suggest that these are sensitive indicators that can be used to estimate the SES effect on individual’s risk of hospital utilization (11, 12). Housing as an important social determinant of health is well acknowledged (13, 14). Disadvantaged housing condition is associated with poor health including a higher prevalence of infectious diseases, injuries, and chronic diseases (13). Poor housing conditions, such as poor sanitation, crowding, poor indoor air quality, and inadequate ventilation, contribute to communicable diseases, chronic diseases, injuries, and poor health outcomes. It is also closely related to low SES and social instability which by itself predisposes to malnutrition, lack of access to affordable health care, and poor health (15).

However, the use of area-level estimates of the SES effect may not be effective in communities where there is no clear geographical segregation of housing along SES lines. In Singapore, for example, housing policy had been used as a social engineering tool to prevent the formation of ghettos (16). There remain markers of low SES associated with housing that can be used to identify individuals at risk. A survey by George et al. suggests that patients staying in one to two room public rental flats in Singapore have higher health-care utilization (17), although the study was limited by the small sample size and self-reporting of outcomes. Nevertheless, residence in public rental housing appears to be as promising as an area-level measure of SES in Singapore.

Singapore is an affluent Asian economy (gross domestic product of about S$73,000) with a multi-ethnic population of 5.6 million people. In Singapore, home ownership is a key local indicator of SES (18). For the needy, heavily subsidized public rental housing is available from the Housing Development Board. About 5.3% of resident households in Singapore reside in public rental housing in 2014 (19). To be eligible for public rental housing at highly subsidized rates, the total household gross income must be very low and not exceed S$1,500 per month. Those staying in public rental housing earn S$2,313 per month, while the national median household income is S$8,290 per month (20).

Therefore, public rental housing is a good area-level measure of SES in Singapore. Using public rental housing as a marker of low SES in this study, we sought to study the impact of residence in public rental housing on readmission risk and hospital services utilization [admissions, emergency department (ED) attendances, specialist outpatient clinic (SOC) attendance] in Singapore.

## Materials and Methods

### Study Design

This is an observational study using retrospective 2014 data from Singapore General Hospital (SGH) electronic health records (EHRs).

### Setting and Study Population

Ministry of Health Singapore created six regional health systems (RHSs) in 2011, each being responsible to adopt a population health approach and integrate care for a specific geographic region. The Singapore Health Services (SingHealth) RHS is the largest health-care cluster in Singapore providing care for the South-central part of Singapore. Each RHS is anchored by an acute hospital, supported by a community hospital providing stepdown and rehabilitation care and complete with linkages to primary care and long-term care services in the region. SGH is the flagship hospital of the SingHealth RHS and the largest tertiary hospital in Singapore with 37 clinical specialties and 88,000 inpatient admissions each year.

To be eligible for inclusion in our study, patients must have at least one clinical encounter (admission or ED visit) to SGH in 2014. Patients who died in 2014 or are non-residents were excluded from analysis. We excluded patients who died as they would not have the whole of 2014 to accumulate readmissions and/or ED visits for our study outcomes. Similarly, we excluded patients who resided in areas where SGH is not the primary hospital, as these patients are likely to be cared for by a different RHS. Similarly, patients discharged to long-term residential care facilities were excluded as these could be located in geographical areas outside of the region served by the SingHealth RHS and confound the frequency of subsequent hospital admissions.

### Data Collection, Variables, and Outcome Measures

Patient-related data (demographics, clinical data, health services utilization, and mortality) were extracted in a de-identified format from the hospital’s enterprise analytics platform.eHINTS (Electronic Health INTelligence System) is the enterprise data repository and analytical platform, which serves the analysis and reporting needs of finance, operations, and clinical users in SingHealth. It integrates financial, operational, and clinical data from multiple systems, including the EHR, across SingHealth institutions to provide users with comprehensive information. Its analytical tools, “self-service” drill down capabilities and location analytics enables faster and more efficient reporting and analysis.

We adjusted for the variables that can affect readmission risk and hospital utilization based on the previous literature to evaluate the independent association of public rental housing with readmission risk and hospital utilization (21–23).

Patient demographics extracted include age, gender, marital status, race, and admission ward class. Our study used residence in public rental housing (housing development board one and two room rental flats) as an indicator of SES in Singapore. Postal codes are unique for each apartment block in Singapore, and all flats in the same block share the same postal code instead of individual codes. In our dataset, public rental housing is recoded based on postal codes published by the Singapore Government (18). Hospital admission payment mode and medical insurance variables, including coinsurance, Medishield, Medical Claims Proration System (MCPS), Medifund, others, and self-payer, were also extracted.

Clinical data extracted included major diseases listed under the Charlson Comorbidity Index (CCI) and the number of surgical procedures, length of stay of index admission, and urgency of index admission. The CCI consist of 17 major diseases, including myocardial infarction, congestive heart failure, cerebrovascular accident, dementia, and diabetes (24). These diseases were identified using International Classification of Diseases (ICD-10) codes in any primary or secondary diagnosis fields dating back to 1 year preceding the index admission. The CCI score was computed for each patient using the using the comorbidities.icd10 package (https://github.com/gforge/comorbidities.icd10) in R version 3.2.3 (R Foundation, Vienna, Austria) during preprocessing of the data. The LACE score (length of stay, acuity of admission, CCI, and ED visits in past 6 months) derived in Ontario, Canada is calculated by summing the points of the above four variables (25). To examine the effect of high risk clinical conditions, we derived the diagnoses of congestive cardiac failure, cerebrovascular disease, chronic obstructive pulmonary disease, and malignancies using ICD-10 codes (26) in our dataset. Depression or other mental conditions were inferred by identifying patients on anti-depressant treatment (mirtazapine, fluvoxamine, escitalopram, and fluoxetine) and anti-psychotic treatment (haloperidol, olanzapine, and risperidone) based on the discharge medications in the EHR. Prior health-care utilization (number of admissions, number of ED visits, and number of SOC visits) in the preceding 1 year were also extracted.

The outcomes studied were (1) readmission within 30 days of discharge; (2) readmission within 15 days of discharge; (3) frequent admissions in 2014, defined as a patient with three or more admissions in 1 year by the Ministry of Health Singapore with an average health-care cost per year approaching S$30,000 (27); (4) frequent ED attendances in 2014, defined as a patient with four or more ED attendances on 1 year by the Ministry of Health Singapore; and (5) frequent outpatient clinic attendance in 2014. These outcomes were chosen as they represent patients who are frequent users of the health-care system. This study was approved by the Singapore Health Services (SingHealth) Centralized Institutional Review Board (CIRB Ref: 2015/2076) with a waiver of patient consent.

### Statistical Analysis

The demographics, clinical, and health services utilization data were compared between patients residing in rental housing and those who are not. Chi-square test or Fisher exact test was used to compare categorical factors associated with staying in rental housing where appropriate. Independent samples *t*-test was used to compare normally distributed continuous variables and Mann–Whitney *U*-test was used to assess the continuous variables, which are not normally distributed. We assessed the independent relationship between residing in rental housing and hospital reutilization and readmission outcomes after adjusting for the demographic and clinical characteristics documented in the previous literature (21–23, 28). We selected the included variables in the multivariate analyses based on whether they were significant at *p* < 0.05 in the univariate analysis and used a backward step-wise procedure in the multivariate models. The model selection was also assessed using the Akaike’s Information Criterion (AIC) and Bayesian information criterion (BIC). Modified Poisson regression model with a robust error variance was used to estimate the relative risk (RR) of the outcomes, since studies have shown that odds ratio (OR) from logistic regression models can substantially overestimate the RR when the outcome is common with the incidence of 10% or more (29, 30). We used the negative binomial model to assess the relationship between rental housing and frequent SOC attendance outcomes since the counts of frequent outpatient attendance were Poisson distributed and showed over-dispersion. Statistical significance was set at *p* < 0.05.

This study defined frequent hospital admissions as three or more hospital admission in 1 year (27). Frequent attenders to ED were defined as patients who attended the ED four or more times a year (31). All data analyses were performed with Stata version 13 (StataCorp, College Station, TX, USA).

## Results

A total of 14,457 unique patients fulfilled both inclusion and exclusion criteria were analyzed, and 2,163 patients (15.0%) were rental housing residents. Table 1 shows the characteristics of included patients stratified by staying at rental housing. The majority of patients were Chinese and those with unknown marital status. Patients who stayed at rental housing were associated with younger age, male gender, non-Chinese ethnicity, requiring financial assistance, chronic obstructive pulmonary disease, usage of anti-depressant and psychotic medication, longer length of hospital stay during the index admission, higher LACE scores, higher proportion of patients with comorbidities, readmission within 15 and 30 days, frequent hospital admissions (three or more), and frequent ED visits (four or more). But, residing in rental housing was associated with fewer SOC visits.

**Table 1:** **Patient characteristics and their association with residence in public rental housing**.

	Reside in rental housing (n = 2,163)	Did not reside in rental housing (n = 12,294)	Total (n = 14,457)	p-value
Age, median (IQR)	62 (49–74)	65 (52–75)	14,457	<0.001*
Gender				<0.001*
Female (%)	970 (44.85)	6,442 (52.4)	7,412	
Male (%)	1,193 (55.15)	5,852 (47.6)	7,045	
Ethnicity				<0.001*
Chinese (%)	1,332 (61.58)	10,217 (83.11)	11,549	
Indian (%)	266 (12.3)	1,086 (8.83)	1,352	
Malay (%)	504 (23.3)	729 (5.93)	1,233	
Others (%)	61 (2.82)	262 (2.13)	323	
Marital status				<0.001*
Married (%)	380 (17.57)	2,615 (21.27)	2,995	
Single (%)	133 (6.15)	604 (4.91)	737	
Divorced/widowed/separated (%)	45 (2.08)	172 (1.4)	217	
Others (%)	4 (0.18)	10 (0.08)	14	
Unknown (%)	1,601 (74.02)	8,893 (72.34)	10,494	
Coinsurance	24 (1.11)	627 (5.1)	651	<0.001*
Medishield	455 (21.04)	4,292 (34.91)	4,747	<0.001*
MCPS	40 (1.85)	721 (5.86)	761	<0.001*
Medifund	115 (5.32)	36 (0.29)	151	<0.001*
Medisave	1,817 (84)	10,434 (84.87)	12,251	0.305
Others	66 (3.05)	340 (2.77)	406	0.458
Self-payer	688 (31.81)	3,457 (28.12)	4,145	<0.001*
Number of surgical procedures, mean (SD)	0.38 (0.77)	0.48 (0.81)	14,457	<0.001*
Length of stay of index admission in 2014, median (IQR)	3 (1–5)	2 (1–5)	14,454	<0.001*
Index admission was urgent (%)	1,856 (85.81)	8,585 (69.83)	10,441	<0.001*
Charlson Comorbidity Index score, median (IQR)	0 (0–0)	0 (0–0)	14,454	<0.001*
Mean (SD)	0.25 (0.77)	0.15 (0.60)		
ED visits (6-month before index admission), median (IQR)	0 (0–1)	0 (0–1)	14,457	<0.001*
Mean (SD)	0.61 (1.36)	1.19 (2.82)		
LACE score, median (IQR)	6 (5–8)	5 (4–7)	14,457	<0.001*
Hospital admissions (1 year before index admission), median (IQR)	0 (0–2)	0 (0–1)	14,457	<0.001*
Mean (SD)	1.11 (2.34)	1.56 (3.52)		
Specialist clinic visits (1 year before index admission), median (IQR)	1 (0–6)	2 (0–7)	14,457	<0.001*
Mean (SD)	4.45 (8.49)	5.11 (8.59)		
ED visits (1 year before index admission), median (IQR)	0 (0–1)	0 (0–1)	14,457	<0.001*
Mean (SD)	0.36 (0.84)	0.67 (1.62)		
Readmission within 30 days (%)	309 (14.29)	1,309 (10.65)	1,618	<0.001*
Readmission within 15 days (%)	199 (9.2)	890 (7.24)	1,089	0.001*
Frequent hospital admission (three or more in 1 year) (%)	339 (15.67)	1,284 (10.44)	1,623	<0.001*
Frequent ED attendance (four or more in 1 year) (%)	286 (13.22)	676 (5.50)	962	<0.001*
Specialist outpatient clinic visits in 2014, median (IQR)	0 (0–2)	0 (0–4)	14,457	<0.001
Mean (SD)	1.89 (4.21)	3.11 (5.97)		
Congestive cardiac failure (%)	53 (2.45)	232 (1.89)	285	0.082
Cerebrovascular disease (%)	11 (0.51)	64 (0.52)	75	0.943
Chronic obstructive pulmonary disease (%)	61 (2.82)	123 (1)	184	<0.001*
Any malignancy	5 (0.23)	26 (0.21)	31	0.855
On anti-depressant	98 (4.53)	340 (2.77)	438	<0.001*
On anti-psychotic	40 (1.85)	145 (1.18)	185	0.011*

*IQR, interquartile range; ED, emergency department; LACE, length of stay, acuity of admission, Charlson Comorbidity Index score, ED visits in the previous 6 months*.

*p < 0.05 (statistically significant).

After adjusting for demographics and clinical conditions known to affect readmission risk and hospital services utilization, the RR of readmission within 15 and 30 days associated with residence in public rental housing was 1.19 (1.02–1.39), *p* = 0.029 and 1.27 (1.12–1.43), *p* < 0.001, respectively (Table 2). Patients staying in public rental housing have a 19 and 27% higher odds of being readmitted within 15 and 30 days, respectively.

**Table 2:** **The impact of residing in public rental housing on readmission risk and hospital services utilization**.

Outcomes	Residing in public rental housing OR (95% CI)^a^	*p*-value
Readmission within 15 days	1.19 (1.02–1.39)	0.029*
Readmission within 30 days	1.27 (1.12–1.43)	<0.001*
Frequent hospital admission (three or more)	1.27 (1.14–1.43)	<0.001*
Frequent ED visits (four or more)	1.40 (1.21–1.61)	<0.001*
Specialist outpatient clinic attendance	0.92 (0.83–1.02)	0.112

*^a^Adjusted for age, gender, ethnicity, marital status, hospital admission payment mode, Charlson Comorbidity Index score, number of surgical procedures, length of stay, number of hospital admission (1 year before index admission), specialist outpatient clinic visits (1 year before index admission), emergency department visits (1 year before index admission), history of malignancy, usage of anti-depressant drugs, usage of anti-psychotic drugs, LACE score, chronic obstructive pulmonary disease, congestive cardiac failure, and cerebrovascular disease*.

*p < 0.05 (statistically significant).

The RR of frequent hospital admissions and frequent ED attendances associated with residence in public rental housing was 1.27 (1.14–1.43), *p* < 0.001 and 1.40 (1.21–1.61), *p* < 0.001, respectively (Table 2). Patients staying in public rental housing have a 27 and 40% higher risk of being a frequent hospital admitter and frequent ED attendee, respectively. Staying in public rental housing showed a 8% lower risk per one SOC visit, but the result was statistically non-significant, 0.92 (0.83–1.02), *p* = 0.112 (Table 2).

## Discussion

We set out to study the association of public rental housing as an area-level measure of SES with readmission risk and hospital services utilization. We found that residents in public rental housing have higher risk of 15-day, 30-day readmissions and being a frequent admitter and frequent ED attendee, even after adjustment for demographics and clinical factors. The consistent trend across four outcomes shows a strong, consistent link between residence in public rental housing and increased hospital utilization. Although the Singapore model of housing, health-care structure, and financing is unique from other countries, our findings add to existing literature on an association between low SES and increased hospital utilization. Our study also highlights the importance and possibility of building healthier communities beyond hospital walls to reduce utilization of hospital services.

Singapore offers universal health-care coverage but adheres to the philosophy of individual responsibility where citizens are expected to make co-payment on top of government subsidies. This co-payment amount, in turn, may be reduced further by three major financing schemes, such as Medisave, MediShield Life, and Medifund. Medisave is a mandatory health savings account and can be withdrawn to pay the hospital bills of the account holder and immediate family members. Medishield Life is a basic health insurance plan, which helps to pay for large hospital bills and selected costly outpatient treatments, such as dialysis and chemotherapy for cancer. The premiums are subsidized by the government and can be paid for through the patients’ own Medisave account. Finally, Medifund is a safety net that is designed as a funding of last resort for patients who are needy and unable to pay despite optimal use of the other financial support schemes. This is to ensure that no citizen is denied appropriate essential health care. In a ranking of health-care systems of the world with a strong emphasis on access, equality and health-care financing, Singapore was ranked 6th out of 191 countries (32). Therefore, affordability and access to health care are unlikely to have a major impact on health-care utilization pattern of patients with low SES in Singapore.

In medicine, there is a preoccupation with developing health-care policies targeting medical determinants of health, such as genetic predisposition and disease-specific risk factors. There is now increasing awareness of the importance developing health policies in the context of the social environment and the social determinants of health (33). Singapore’s public housing policy is considered to be one of the most progressive in the world with 82% of its population living in high quality public housing. It has the highest home ownership rate in the world at 93%. Even under such a favorable setting, housing remains a strong determinant of health (34). The implications of our findings are that policy makers need to engage rental housing residents to obtain a deeper insightful understanding of the social circumstances, health seeking behavior, and health literacy. Addressing these actionable risk factors may reduce unnecessary health-care utilization.

The reasons behind poor outcomes in patients residing in public rental housing are many. Disadvantaged housing condition is both a direct cause of poor health through poor household conditions, such as sanitation, overcrowding, and poor indoor air quality, contributing to communicable diseases and exacerbations of chronic illnesses and a marker of low SES and social instability that compromises access to health care (13, 35). In our study, patients staying in public rental housing in Singapore have more comorbidities as indicated by their higher CCI scores. They are more likely to be on anti-depressant treatment, consistent with findings from Wee et al. (36). Interestingly, they do not appear to be more likely to have cancer, congestive heart failure, or stroke, but the prevalence of chronic obstructive pulmonary disease is higher and statistically significant. Unfortunately, we do not have data on smoking habit of the subjects, which might provide possible explanation of the observed difference. On the other hand, this could also mean that housing has a direct impact on the prevalence and optimal control of COPD. Indoor air pollution had been shown to be associated with chronic lung diseases (37). Other studies had shown that COPD as a chronic disease is most sensitive to the effects of social deprivation (38, 39). This is consistent with our findings.

In our study, patients in public rental housing are more likely to be single, widowed, or divorced. There is an over-representation of minority ethnic groups in comparison to the general population. These are factors that are known to be associated with poorer health outcome. Housing therefore may be a good composite indicator of socioeconomic disadvantage and explains its importance as a social determinant of health. Health-care policies aimed at rationalizing the use of hospital resources must take socioeconomic factors into consideration. Data about housing are unobtrusive and readily available in most hospital administrative datasets. Our study showed that housing can be associated with high health-care needs and utilization of hospital resources. This knowledge can help us to allocate resources and develop medical and social interventions to help such individuals and improve programs aimed at optimizing cost effectiveness of health-care systems.

For patients of low SES, the hospital discharge plan prescribed by health-care workers are often confusing, unrealistic, and impractical in the face of significant socioeconomic constraints and more pressing needs that such patients may face in their daily life (40). In-depth interviews by Kangovi et al. found that residents in high poverty areas were unable to access the care and accommodations needed to cope with post-hospital frailty. As a result, medical disability was amplified by socioeconomic disability (41).

We found it interesting that rental housing residents in our cohort have a different pattern of utilization of health-care resources. They visited fewer specialists in the outpatient setting. There are few studies that evaluated the impact of SES on outpatient specialist use and Filc et al. reported that patients with higher SES visited more specialists (4), which is similar to our results. Although our data do not include primary care utilization, a study by Wee et al. found that western-trained physicians are not the first choice of lower income Singaporeans seeking primary care. Many preferred alternative medicine or self-medication (42). Barriers, such as poor knowledge, costs, and characteristics of primary care practices, must be addressed if better outcomes were to be achieved.

### Limitations

Although our study was carefully prepared, there were some limitations. First, our dataset did not include patients who could have utilized only outpatient services in our health system. However, these patients are low risk, and our aim was to study the association of housing with readmission risk after an index hospital admission and subsequent hospital services utilization. Second, variables in our dataset are restricted to those routinely collected in the EHR and administrative databases. As such, the granularity of social determinant variables is restricted to housing and payment modes, and functional status was not available. By excluding patients who were discharged to long-term institutional care, we likely minimized gross differences in function between rental housing and non-rental housing patients. Third, due to the retrospective nature of the study, we were unable to prove a causal association between rental housing and health outcomes. In addition, although our study was conducted at the largest health system in Singapore, we were unable to account for readmissions to other health systems. We minimized such bias by excluding patients who stay in geographical locations served by other health systems. Finally, although our study adds to existing literature that housing is a good proxy marker of SES and is independently associated with increased hospital services utilization, and this should be interpreted within the context of Singapore’s unique housing and health-care policies.

## Conclusion

Our study found that public rental housing as an area-level measure of SES in Singapore is independently associated with increased readmission risk and being a frequent hospital admitter and ED user. Policy makers and the health system need to engage rental housing residents to obtain a deeper insightful understanding of the social circumstances, health seeking behavior, and health literacy. Intervening in these modifiable risk factors may reduce unnecessary health-care utilization.

## Ethics Approval

This study was approved by the Singapore Health Services (SingHealth) Centralized Institutional Review Board (CIRB Ref: 2015/2076) with a waiver of patient consent.

## Author Contributions

Conceived and designed the study: LL and KL. Performed the study: LL, WW, and NL. Analyzed the data: WW and LL. Interpreted the results: LL and KL. Wrote the paper: LL, WW, and KL. Principal Investigator of this study and supervised this study: LL. Revised the paper critically and give final approval for publication: all authors.

## Conflict of Interest Statement

The authors declare that the research was conducted in the absence of any commercial or financial relationships that could be construed as a potential conflict of interest.

## Abbreviations

CCI, Charlson Comorbidity Index; ED, emergency department; RHS, Regional Health System; SES, socioeconomic status; SGH, Singapore General Hospital.
